# Pathway Analyses Implicate Glial Cells in Schizophrenia

**DOI:** 10.1371/journal.pone.0089441

**Published:** 2014-02-24

**Authors:** Laramie E. Duncan, Peter A. Holmans, Phil H. Lee, Colm T. O'Dushlaine, Andrew W. Kirby, Jordan W. Smoller, Dost Öngür, Bruce M. Cohen

**Affiliations:** 1 Department of Epidemiology, Harvard School of Public Health, Boston, Massachusetts, United States of America; 2 Psychiatric and Neurodevelopmental Genetics Unit (PNGU), Massachusetts General Hospital, Boston, Massachusetts, United States of America; 3 Center for Human Genetic Research, Massachusetts General Hospital, Boston, Massachusetts, United States of America; 4 Stanley Center for Psychiatric Research, Broad Institute of MIT and Harvard, Cambridge, Massachusetts, United States of America; 5 Department of Psychiatry, Harvard Medical School, Boston, Massachusetts, United States of America; 6 MRC Centre for Neuropsychiatric Genetics & Genomics, Cardiff University, Cardiff, United Kingdom; 7 Analytic and Translational Genetics Unit (ATGU), Massachusetts General Hospital, Boston, Massachusetts, United States of America; 8 Schizophrenia and Bipolar Disorder Program, McLean Hospital, Belmont, Massachusetts, United States of America; 9 Shervert Frazier Research Institute, McLean Hospital, Belmont, Massachusetts, United States of America; University of Illinois at Chicago, United States of America

## Abstract

**Background:**

The quest to understand the neurobiology of schizophrenia and bipolar disorder is ongoing with multiple lines of evidence indicating abnormalities of glia, mitochondria, and glutamate in both disorders. Despite high heritability estimates of 81% for schizophrenia and 75% for bipolar disorder, compelling links between findings from neurobiological studies, and findings from large-scale genetic analyses, are only beginning to emerge.

**Method:**

Ten publically available gene sets (pathways) related to glia, mitochondria, and glutamate were tested for association to schizophrenia and bipolar disorder using MAGENTA as the primary analysis method. To determine the robustness of associations, secondary analyses were performed with: ALIGATOR, INRICH, and Set Screen. Data from the Psychiatric Genomics Consortium (PGC) were used for all analyses. There were 1,068,286 SNP-level p-values for schizophrenia (9,394 cases/12,462 controls), and 2,088,878 SNP-level p-values for bipolar disorder (7,481 cases/9,250 controls).

**Results:**

The Glia-Oligodendrocyte pathway was associated with schizophrenia, after correction for multiple tests, according to primary analysis (MAGENTA p = 0.0005, 75% requirement for individual gene significance) and also achieved nominal levels of significance with INRICH (p = 0.0057) and ALIGATOR (p = 0.022). For bipolar disorder, Set Screen yielded nominally and method-wide significant associations to all three glial pathways, with strongest association to the Glia-Astrocyte pathway (p = 0.002).

**Conclusions:**

Consistent with findings of white matter abnormalities in schizophrenia by other methods of study, the Glia-Oligodendrocyte pathway was associated with schizophrenia in our genomic study. These findings suggest that the abnormalities of myelination observed in schizophrenia are at least in part due to inherited factors, contrasted with the alternative of purely environmental causes (e.g. medication effects or lifestyle). While not the primary purpose of our study, our results also highlight the consequential nature of alternative choices regarding pathway analysis, in that results varied somewhat across methods, despite application to identical datasets and pathways.

## Introduction

The molecular etiologies of schizophrenia and bipolar disorder are not yet understood, hindering efforts to develop novel pharmacological treatments. Accordingly, pharmaceutical companies have drastically reduced investment in psychiatric drug discovery [1]. In order to develop therapeutics for the majority fraction of patients who still do not respond adequately – or at all – to currently available treatments, new insights into the molecular etiology of these and other psychiatric disorders are needed.

Both schizophrenia and bipolar disorder are known to be highly heritable, with estimates of 81% for schizophrenia and 75% for bipolar disorder [2]. Groundbreaking discoveries about specific genetic risk variants have occurred in the last few years. In schizophrenia, both copy number variant (CNV) and single nucleotide polymorphism (SNP) associations have been identified using genome-wide methodologies [2], [3]. Genome-wide association studies (GWAS) have also identified SNPs associated with bipolar disorder [4]. Of great importance regarding the number and distribution of genetic risk factors, polygenic analyses suggest that both disorders are influenced by thousands of risk loci [3] distributed across the genome, and further – that many loci are shared across schizophrenia and bipolar disorder [5]. Thus, despite these advances, efforts to understand the biological processes and pathways affected by genetic risk variants are just beginning. Further, as individual genes each only contribute modestly to risk, but genotype as a whole is highly determinant of psychotic illness, further studies of functionally related gene groups and their interactions seem especially promising. Results of such analyses may identify new targets for study and intervention.

### Pathway analyses

One analytic approach designed to provide information about the relevant biology implicated by genetic associations is known as ‘pathway analysis’. In contrast to typical GWAS – which evaluates the significance of association to phenotype for each SNP *individually* – pathway analyses are designed to determine the significance of association between phenotype and variants in a *group of genes* related by function. The pathway is the unit of analysis instead of each individual SNP, one by one. Regarding terminology, it is often technically more correct to say ‘gene set’ analyses because the term ‘pathway’ implies a more specific relationship among genes. However, throughout this manuscript, in keeping with common practice, we refer to ‘gene set analyses’ as ‘pathway analyses’.

It is important to note that while these pathways contain genes that are critically important for the specified cells or processes in question, some of the same genes may be relevant in varying degrees for other cells and processes. Such sets of genes are an appropriate analysis unit for disorders such as schizophrenia and bipolar disorder that have multifactorial inheritance and are likely due to multiple, often subtle, changes in large numbers of genes. The combined effect of numerous disrupted genes impacting a specific cell type or function is exactly what pathway analysis can reveal, and the specific genes involved and their precise functions and relationships can be refined in future studies. Of course, the results may point to anomalies in other cell types or other processes that share some of the same genes, and these other processes can also be tested for disease association in further studies.

Clearly, the power of analyzing by pathways depends on accurate identification of genes that contribute to the specific pathophysiology of the illness studied. Past pathway analyses of schizophrenia have implicated synaptic, neuronal cell adhesion, and membrane scaffolding gene sets [6], [7]. The goal of the present study is to test, in large GWAS datasets, hypotheses about the involvement of groups of genes determining glial, mitochondrial, and glutamate function in schizophrenia and bipolar disorder. These pathways were chosen based on convergent evidence of these elements being abnormal in schizophrenia and bipolar disorder, as summarized below. Details about pathway construction are given in the methods section, and lists of genes within pathways are provided in the data supplement (**Data S1**).

#### Glial Pathways

Both major subclasses of glial cells, oligodendrocytes and astrocytes, may be altered in psychotic disorders (for reviews of the findings cited below [8]–[10]). *In vivo* brain imaging and *post-mortem* studies consistently report somewhat reduced and disorganized white matter in psychosis, with these abnormalities being greater in schizophrenia, but also seen in bipolar disorder. Gene expression studies point to oligodendrocytes, the cells responsible for myelination and the maintenance of white matter, as functionally abnormal in psychotic disorders. Dysfunction in oligodendrocyte development, location, or function (i.e. ability to make myelin) might be the cause of white matter abnormalities in schizophrenia. Some evidence from brain imaging and postmortem studies also suggest abnormalities in the number or density of astrocytes, which perform crucial tasks in stabilizing glutamatergic synapses, both in bipolar disorder and schizophrenia. Of likely relevance, one of the most consistent findings in psychiatric post mortem research is a reduced density of glia in prefrontal cortex in mood disorders, including bipolar disorder. Currently it is not definitively known if the missing cells are astrocytes or oligodendrocytes.

#### Mitochondrial Pathways

The brain requires twenty times the energy of the rest of the body (per unit weight) to maintain its functions [11]. Energy expenditure supports maintenance of ionic gradients across neural membranes and the recycling and metabolism of potentially toxic neurotransmitters, including glutamate (see below). Even a modest reduction in energy production can lead to neuronal dysfunction by impairing the metabolism of key compounds or reducing the production and recovery of electrical gradients across the membranes [12]. Evidence consistent with an abnormality of energy production has been reported both for schizophrenia (for reviews of the evidence listed below [13]–[16]) and bipolar disorder (for reviews of the evidence below [17]–[20]). Specifically, the evidence suggests alterations in the function of mitochondria, which are responsible for most cellular energy production. Findings in schizophrenia include reduced expression of genes and proteins associated with mitochondrial function, both in brain post mortem and in peripheral cells. *In vivo* observations include low metabolic activity and low levels of the high-energy molecule ATP in brain. Also, a reduced density of mitochondria in peripheral cells and in synapses, specifically, has been reported for schizophrenia and for psychotic disorders in general. Similar evidence in bipolar disorder includes reduced expression of genes associated with mitochondrial function, both in post mortem brain and peripheral cells. This is especially true for cells under stress. In addition, *in vivo* brain imaging studies report elevated lactate, suggesting reduced oxidative phosphorylation, and a slow recovery of the high-energy storage molecule phosphocreatine after depletion by regional brain activation. Altered mitochondrial shape, which implies altered function, has also been observed in both brain and peripheral cells in bipolar disorder.

#### Glutamate Pathway

Glutamate is the major excitatory neurotransmitter in the brain. This role, its effects on cell growth and its toxic properties, have all led to proposals that abnormalities of glutamate underlie psychotic and mood disorders (For reviews of the evidence cited here [21]–[24]). Psychosis and mood dysregulation can be induced by drugs that antagonize glutamate NMDA receptors. More recently, drugs have been developed to modulate activity at either NMDA or metabotropic glutamate receptors and reduce symptoms of schizophrenia and depression. Post mortem studies suggest altered activity of glutamate synthetic and catabolic enzymes both in schizophrenia and bipolar disorder. Brain imaging studies also reveal abnormal levels and turnover of glutamate and its metabolites in both disorders. Most recently, genetic studies have reported the association of genes for glutamatergic neurotransmission and psychotic disorders [24].

### Present Study

The origin of the abnormalities noted above, and even whether they are causal or epiphenomenal, is not known. In the present study, we examine evidence for association between genes in these biologically relevant pathways and both schizophrenia and bipolar disorder. Although this is not a study of the pathway methods, themselves, we did have to address a methodological challenge in choice of the pathways used: while alternative pathway analysis methods are conceptually similar, in that they are designed to provide composite metrics of association for sets of genes, they provide results that are only modestly correlated with one another. This is true because alternative pathway analysis methods aggregate and evaluate SNP-level information in different ways. Moreover, the ‘best’ pathway analysis method is not known, nor is there likely a single best method, as different methods will be better suited to detecting the as-yet-unknown genetic architectures underlying different psychiatric disorders. Thus, we picked one method, MAGENTA [25], for the primary analysis because it was the first method available to our group. Through subsequent collaborations, we then applied three additional pathway analysis methods: ALIGATOR [26], INRICH [27], and Set Screen [28]. We expected that results would vary somewhat across the four pathway analysis methods, but knew that any degree of consensus would be helpful in guiding future work. The use of multiple measures is a check on the robustness of any findings observed by the primary analysis and could provide, in an exploratory fashion, other leads on relevant pathways for future study.

## Methods

### Genotypic data for Schizophrenia and Bipolar Disorder

Publically available Psychiatric Genomics Consortium (PGC) data were downloaded for schizophrenia [29] and bipolar disorder [4]. The specific files used were pgc.scz.full.2012-04.txt and pgc.bip.full.2012-04.txt, from https://pgc.unc.edu/Sharing.php#SharingOpp. Publically available PGC datasets contain the primary but not replication samples, and have the following sample sizes for schizophrenia (9,394 cases/12,462 controls) and bipolar disorder (7,481 cases/9,250 controls). These files contain p-values for imputed SNPs and were filtered for info scores >0.8, yielding 1,068,286 SNP-level p-values for schizophrenia and 2,088,878 SNP-level p-values for bipolar disorder. Gene boundaries were defined as 35 kilobases upstream of genes and 10 kilobases downstream of genes, chosen to capture most promoter regions and cis-eQTLs [30]. All coordinates were hg18 (Human Genome build 18). Finally, the MHC region (defined here as chromosome 6, bases 25,000,000–35,000,000) was removed prior to analysis given extensive linkage disequilibrium (LD) in the region. However, none of the genes in pathways tested in this report overlapped the HLA region, so this had no effect on our results. No other regions or SNPs were removed from analysis.

**Figure 1:**
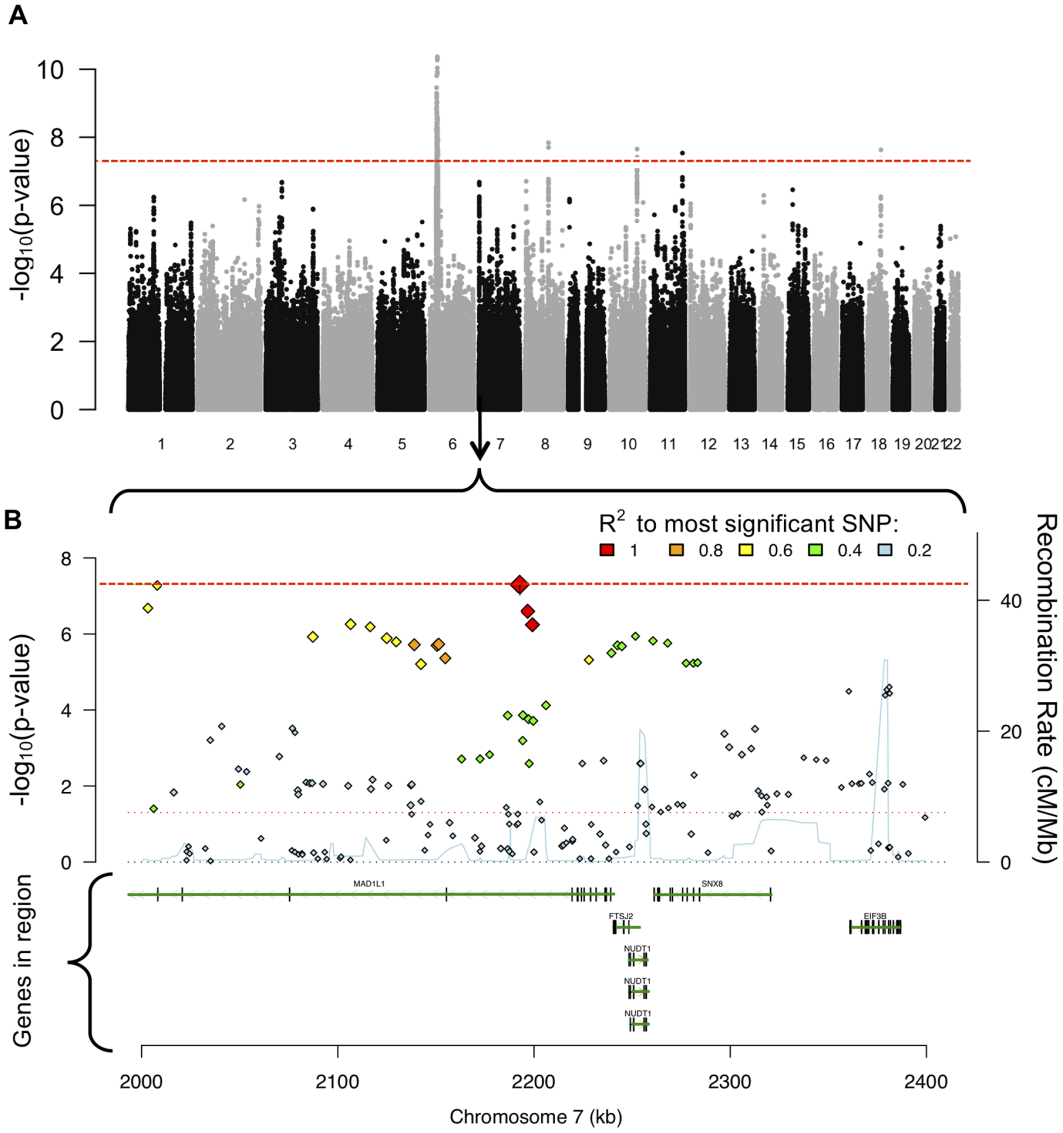
Manhattan plots illustrating data use decisions in pathway analyses. Figure 1 utilizes a Manhattan plot of 22 chromosomes (A) and a detailed Manhattan plot for a region of chromosome 7 (B) to illustrate differences in pathway analytic methods. One major distinction among pathway analysis methods designed for GWAS data concerns the use of raw data (i.e. individual level genotypes) versus summary statistics (i.e. p-value, odds ratios, betas, etc. per SNP). For example, the paper recently published by Goudriaan et al (2013) used raw genotype data. In contrast, the four methods used in this report all use SNP-level summary statistics as input data. Figure 1A is a 'Manhattan plot, in which each point represents one SNP. The x-axis denotes chromosomal position and the y-axis denotes significance of each SNP's association to the phenotype (units of -log_10_p-value). The horizontal red line denotes genome-wide significance (p<5×10^−8^). In Figure 1B, a section of the Manhattan plot on chromosome 7 is expanded, and the location of genes is given in the lower portion of section 1B. As seen in 1B, individual genes may contain many SNPs (colored diamonds in figure). Three of the pathway analytic methods used in this report use a ‘best SNP per gene/region’ approach, meaning that they ‘count’ only the most significant SNP in a gene or region of interest (methods MAGENTA, ALIGATOR, and INRICH). In contrast, Set Screen utilizes information from all SNPs within a gene or region of interest in the calculation of pathway-level statistics. Another aspect of the design of pathway analyses is illustrated in 1B: correlation among SNPs. Due to the haplotype structure of chromosomes, many genetic variants are correlated with one another, and are said to be in ‘linkage disequilibrium’ or ‘LD’. Thus, each method used in this report has analytic procedures for handling LD, so that correlated signals are not inappropriately counted multiple times. As stated in the text, correlation among SNPs is so extensive in the HLA region of chromosome 6, that pathway analytic methods currently exclude this region from analysis. In the past, failure to exclude the HLA from pathway analyses led to the reporting of spurious associations. Finally, we note that assigning of SNPs to genes is an imprecise task. Two complications are overlapping genes and correlated variants that may span many genes. A more daunting challenge is capturing the regulatory elements that impact genes. Such elements may be located far from genes, sometimes even on different chromosomes. Future developments in pathway analytic methods will likely make use of information about tissue and gene-specific regulatory elements (e.g. derived from the ENCODE project), but such information is not currently implemented in these pathway analytic methods.

Figure 1 depicts an example of the type of data used in this report (i.e. SNP-level p-values from GWAS analyses). These ‘Manhattan Plots’ are a standard graphical depiction of p-values (y-axis, in -log_10_ units) by chromosomal position (x-axis). Analytical decision points in pathway analyses (e.g. how to assign SNPs to genes) are described in the caption for Figure 1. Ricopili was used to create the detailed Manhattan plot in Figure 1 B (http://www.broadinstitute.org/mpg/ricopili/).

### Pathway curation (i.e. creation of gene sets for analysis)

Based on prior evidence for the pathways/functional groupings of greatest interest, we identified 262 mitochondria related, 285 glutamate related, and 110 glia related genes sets from the Gene Ontology [31] (GO) and REACTOME [32] databases. Selected gene sets were then combined with genes from the published literature about other mitochondria, glutamate, and glia related genes thought to be involved in the pathophysiology of schizophrenia and/or bipolar disorder – to create the ten gene sets analyzed in this report. We created three superordinate gene sets: Glia, Mitochondria, and Glutamate. The superordinate Glia pathway had two subordinate pathways (Astrocyte and Oligodendrocyte) and the superordinate Mitochondria pathway had five subordinate pathways (Crista, Distribution, Fission, ‘Fission_plus’ with additional genes, and Fusion). There were no subordinate pathways for Glutamate. Gene membership in each of the ten pathways is provided in **Data S1**. A reviewer of the submitted manuscript asked whether, given the substantial overlap in genes used by different cells, any associations between pathways and illness observed in this study might generalize to other cell types. The reviewer suggested studying pathways for lymphocytes, macrophages and hepatocytes. This is an interesting question, especially as abnormalities of peripheral cells, as previously noted, have been observed in psychotic and mood disorders. While the evidence for abnormalities of CNS cells is stronger than that for peripheral cells, we created pathways for lymphocytes, macrophages and hepatocytes, using the public data sets available in Gene Ontology [31] (GO), by methods equivalent to those we used for our primary hypotheses. Membership of genes in these pathways is also provided in the **Data S1.** Analyses of these pathways is necessarily exploratory. Per reviewer suggestion, these exploratory analyses also serve as a negative control against the possibility that genes (such as PPARG and SHH) – which are important for many cell types – might result in a *non-specific* association to oligodendrocytes.

### Pathway analysis methods

We used four distinct and independently developed pathway analysis methods to test hypotheses that the specified mitochondrial, glutamatergic, and glia-related gene sets are associated with schizophrenia and bipolar disorder. The primary analysis method was MAGENTA [25]. To explore sensitivity and robustness of results to different pathway methods, we subsequently ran three additional methods: ALIGATOR [26], INRICH [27], and Set Screen [28]. Each analysis was run separately according to standard protocols for that method. Set Screen requires GC-corrected p-values to avoid spurious pathway associations arising from aggregation of inflated test statistics; therefore lambda-adjusted p-values were used in Set Screen analysis. Default settings were used unless they needed to be altered to conform to the parameters specified above.

By default, MAGENTA employs two thresholds, at ‘95%’ and ‘75%’, which specify the (somewhat arbitrary) threshold above which a gene-level p-value will be deemed ‘significant’. Comparison of the observed number of ‘significant’ genes within a pathway to the expected number of significant genes provides the pathway level p-value (as calculated by the MAGENTA algorithm). The latter threshold of 75% (hereafter, “setting”) captures weaker signals of association than the former, and may be better suited to detecting associations to highly polygenic phenotypes. These two settings were also used for ALIGATOR and INRICH. Set Screen does not have the option of specifying settings, so only one ‘setting’ is reported for Set Screen. These different analytic methods are expected to yield somewhat different results, as they do not use the same assumptions nor model the data in equivalent ways. Rather, they are complementary in seeking possible associations of pathways (gene sets) to illness. Properties of the genome and genetic data which necessitate analytical decisions in pathway analysis are explained in **Figure 1**.

### Multiple testing correction

Though three of the methods (MAGENTA, ALIGATOR, and INRICH) provide corrected p-values or false discovery rates, none of the methods could provide appropriate correction for the use of two settings (95% and 75%, see above) or for the analysis of two different datasets (schizophrenia and bipolar disorder). We use Bonferoni correction within method (e.g. for our primary analysis with MAGENTA), acknowledging that it is conservative given non-independence of genes within pathways and use of data across alternative settings (95% vs. 75%). There is no perfect way to correct for all 140 tests conducted in this report, but we apply FDR [33] control using an R [34] library (‘qvalue’) from Bioconductor [35]. **Tables S2 and Table S3** provide q-values corresponding to all p-values for schizophrenia and bipolar disorder, respectively. Additional details about the application of FDR control in this report are provided in **Methods S1** and **Figure S1**.

## Results

### Primary Analysis

Our primary analyses were performed with MAGENTA. The strongest result was an association between the Glia-Oligodendrocyte pathway and schizophrenia (p = 0.0005). This p-value is significant after conservative Bonferroni correction in our primary (MAGENTA) analysis for 40 tests (10 pathways * 2 settings * 2 disorders). Within the primary analyses, while there were trends of interest, no other pathways were method-wide or nominally significant. Gene-level p-values and other information about the 52 genes in the Glia-Oligodendrocyte pathway are provided in **Table S1**.

Interestingly, the strongest results for the MAGENTA analyses were obtained with the 75% setting, which is effectively a user-defined threshold for gene-level for significance. It is important to note that the use of the alternative (here 75% vs. 95%) threshold is not a more lenient threshold for statistical significance. Rather, adjustment of this setting allows the user to optimize the MAGENTA algorithm for the expected genetic architecture and/or expectations about the data being analyzed. As stated in the MAGENTA script, users may want to rely on the 75% setting for diseases that are known to have highly polygenic genetic architectures. This is the case for schizophrenia.

Another important point pertains to the data being analyzed. For example, in the case of a somewhat underpowered GWAS, p-values for *true risk* alleles may be relatively unimpressive, and odds ratios will not even demonstrate the expected direction of effect on a reliable basis.

Therefore, casting a wider net (via use of a 75% threshold) may allow for better capture of weak signals, which in aggregate are indicative of association. Given what is now known about schizophrenia, both extreme polygenecity and low power to detect the association of individual alleles are likely true. The latter is another reason why larger GWAS samples are needed to provide appropriate replication and clarification of these findings.

### Secondary analysis

Secondary analyses, to test for robustness of the primary findings and as an exploration of alternative pathways to be explored in future studies, were conducted with ALIGATOR, INRICH, and Set Screen. Consistent with the MAGENTA results, the Glia-Oligodendrocyte pathway in the schizophrenia dataset obtained nominally significant p-values using ALIGATOR and INRICH (p = 0.022 and 0.006, respectively); see **Tables 1** & **2**. Further, in both cases the more inclusive setting yielded stronger p-values, indicating more highly polygenic genetic architecture and/or lower power in the schizophrenia GWAS results see **Tables 1** & **2**.

**Table 1:** Schizophrenia pathway p-values by method and setting.

		Primary Analysis		Seondary Analyses				
		MAGENTA		ALIGATOR		INRICH		SS
	Genes	95%	75%	95%	75%	95%	75%	–
**GLIA**	146	0.54	0.06	0.40	0.18	0.29	0.34	0.012#
Glia – Oligodendrocyte	52	0.48	0.0005*	0.21	0.022#	0.17	0.006#	0.14
Glia – Astrocyte	42	0.89	0.24	N/A	0.51	0.74	0.68	0.007#
**MITOCHONDRIA**	74	1.00	0.06	N/A	0.31	0.53	0.66	0.23
Mitochondria – Crista	6	0.22	0.36	N/A	0.60	1.00	0.51	0.011#
Mitochondria – Distribution	7	1.00	0.38	N/A	0.65	1.00	1.00	0.12
Mitochondria – Fission	12	1.00	0.96	N/A	N/A	1.00	1.00	0.035#
Mitochondria – Fission 959	24	0.69	0.54	N/A	0.50	0.34	0.61	0.031#
Mitochondria – Fusion	9	1.00	0.32	N/A	0.44	1.00	0.39	0.08
**GLUTAMATE**	158	0.85	0.45	0.86	0.54	0.46	0.88	0.19

SS  =  Set Screen.

* Denotes method-wide significance, meaning correction for all tests conducted with a given method. For MAGENTA, ALIGATOR, and INRICH, correction is for 40 tests (α = 0.00125 given 10 pathways ×2 settings ×2 disorders). Set Screen correction is for 20 tests given single setting (α = 0.0025).

# Denotes nominal significance (p<0.05).

**Table 2:** Bipolar disorder pathway p-values by method and setting.

		Primary Analysis		Secondary Analyses				
		MAGENTA		ALIGATOR		INRICH		SS
	Genes	95%	75%	95%	75%	95%	75%	–
**GLIA**	146	0.92	0.42	0.68	0.59	0.62	0.86	0.025#
Glia – Oligodendrocyte	52	0.93	0.19	0.41	0.48	0.45	0.64	0.021#
Glia – Astrocyte	42	0.88	0.46	0.46	0.20	1.00	0.80	0.002*
**MITOCHONDRIA**	74	1.00	0.73	0.92	0.74	0.82	0.70	0.51
Mitochondria – Crista	6	1.00	0.37	N/A	0.19	1.00	0.34	0.82
Mitochondria – Distribution	7	1.00	0.17	N/A	0.37	1.00	0.25	0.20
Mitochondria – Fission	12	1.00	0.47	N/A	0.51	1.00	0.51	0.99
Mitochondria – Fission 959	24	0.68	0.67	N/A	0.65	0.24	0.62	0.97
Mitochondria – Fusion	9	1.00	0.87	N/A	0.63	1.00	1.00	0.44
**GLUTAMATE**	158	0.74	0.63	0.78	0.29	0.37	0.17	0.49

SS  =  Set Screen.

*Significant within method, meaning correction for all tests conducted with a given method. For MAGENTA, ALIGATOR, and INRICH, correction is for 40 tests (α = 0.00125 given 10 pathways ×2 settings ×2 disorders). Set Screen correction is for 20 tests given single setting (α = 0.0025).

# Nominal significance (p<0.05).

Across all secondary analyses, ALIGATOR and INRICH results were similar, but not as strong, as the MAGENTA results. In contrast, eight of twenty pathways tested across the schizophrenia and bipolar disorder datasets were at least nominally significant according to Set Screen analysis. For schizophrenia, Set Screen yielded nominally significant associations for a total of five glial and mitochondrial pathways: superordinate Glia (p = 0.012), Glia-Astroctye (p = 0.007), Mitochondria-Crista (p = 0.011), Mitochondria-Fission (p = 0.035), and the larger Mitochondria-Fission_plus pathway (p = 0.031). For bipolar disorder, all three glial pathways were at least nominally significant. The Glia-Astrocyte pathway was method-wide significant (p = 0.002) whereas the Glia-Oligodendrocyte (p = 0.021) and superordinate Glia (p = 0.025) pathways were both nominally significant.

In an effort to determine how many results in this report are likely to be true, we used the FDR control procedures described by Storey, Tibshirani, and Dabney [35], [33]. These procedures allow for the estimation of the number of true results in this report, which is four (4). Q-values corresponding to all p-values (for schizophrenia and bipolar disorder) are provided in **Tables S2** and **Table S3**.

As mentioned, after the completion of our primary and secondary analyses, we explored the association of pathways for peripheral cells, which share some relevant genes with brain cells, with schizophrenia and bipolar disorder. No significant associations were observed. Details are provided in the **Methods S1** and **Table S4**.

### Conclusions

The observed Glia-Oligodendrocyte pathway association to schizophrenia is consistent with an extensive literature confirming white matter abnormalities in schizophrenia [36]. These abnormalities are observed in living patients via brain imaging and also in postmortem analyses. White matter is composed of axons surrounded by myelin produced by oligodendrocytes. If supported in future replication attempts, this finding represents one of the first links for schizophrenia between specific neurobiological findings and specific sets of genes, as assessed in large-scale genetic studies. In all, given the prior evidence implicating oligodendrocytes and the current evidence that genes related to oligodendrocytes are associated with schizophrenia, an oligodendrocyte pathology seems well supported. Future studies might narrow down the most relevant genes and their relationship to the cellular processes that result in disrupted myelination in schizophrenia. It might also be possible to link this pathway and associated abnormalities in oligodendrocytes to the high-level, core features of schizophrenia thought to be based on white matter pathology; for example: disrupted connectivity of regional brain activity and reduced co-ordination of thinking.

The bipolar disorder finding of association to astrocyte development and function genes (observed only with Set Screen) is also intriguing. As noted above, astrocytes may be among the reduced glial cells observed in bipolar disorder [37]. In addition, astrocytes form a tripartite synapse with glutamatergic terminals and postsynaptic densities in the brain and also play a direct role in glutamate metabolism. Thus, astrocyte abnormalities could explain some of the glutamatergic anomalies reported in psychosis and mood disorders. Specifically, some portion of altered glutamatergic signaling may be due to impaired glutamate uptake and recycling by astrocytes, rather than being due to abnormalities of either glutamate release or glutamate receptors (in pre and/or post synaptic neurons).

Finally, in the secondary analyses, we note that Set Screen results suggested nominally significant associations for mitochondrial fission and mitochondrial cristae in schizophrenia. It is important to note the higher likelihood of false positive results in secondary analyses. However, mitochondrial abnormalities have been reported previously in schizophrenia and bipolar disorder. Replication studies with larger samples will provide evidence about the reliability of our findings that specific mitochondrial gene sets may be related to these abnormalities.

The most important consequence of all of these findings and trends may be their biological relevance, specifically their implications concerning etiology and pathophysiology. Psychotic disorders, including schizophrenia, are highly complex by cause and course. They are known to have inherited determinants, but they are also associated with infectious, toxic and traumatic exposure. In addition, life style, life events, and treatment can alter brain biology in those who develop psychosis. For this reason, in vivo brain imaging and post mortem studies are limited in determining if abnormalities observed exist prior to the onset of overt illness or are a consequence or concomitant of illness. These genomic results strongly suggest that some factors, notably those for the function of glial elements in brain, are true risk factors for illness.

### Comparison of results from different pathway analysis methods

We realize that the concordant and discordant results obtained from the separate analytic methods is a phenomenon that is interesting in and of itself. While these differences are expected and further study of the reasons for these differences is an important area of study in the development and interpretation of pathway analysis, such comparisons are beyond the scope of this paper. They would require extensive modeling and are properly the subject of other papers. Our study used established pathway techniques to gain insight into the association of gene sets and psychotic disorders. We used a primary analysis (MAGENTA) and secondary analyses for possible confirmation or expansion of evidence of associations, not to provide a comparison of techniques.

That being said, we found evidence supporting the expectation that selection of pathway analysis method, and the settings used within methods, *is not trivial*. Indeed, we would have reached somewhat different conclusions about the association of these pathways with schizophrenia and bipolar disorder if we had applied only one method. This means that replication attempts of pathway analyses should be conducted using the same method and same settings as the original report if direct comparisons are to be made. However, these results also suggest real value in applying more than one method, so that clues to pathway associations are not missed by the limitations of any one method.

Across the four methods, which set of results is ‘right’? In one sense, they all are. Each of these methods is a valid approach to pathway analysis that creates and evaluates pathway level statistics from the relevant GWAS data. There is undoubtedly variability in the degree to which each method accurately captures genetic signals within pathways, but currently there is no ‘gold standard’. Rather, the suitability of any particular pathway analysis method will vary across pathways and phenotypes because of the (presumably) different underlying genetic architectures (See Figure 1). More and separate research is needed to determine which pathway analysis method is best, for which diseases and which datasets, and what criteria should be used for choosing a method in each case. As noted, resolving these issues will require the use of multiple real data sets as well as simulations and, as such, are well beyond the focus of this report.

However, it is worth noting that the pattern of correlations across results from the four methods is broadly consistent with major similarities and differences among pathway analysis methods used in this report. Whereas Set-screen uses all p-values within all genes in the calculation of pathway-level p-values, ALIGATOR, INRICH, and MAGENTA use a “best-SNP per gene” approach. Further, the latter three methods may be classified as “over-representation” methods, which test the number of genes in a pathway that reach a given significance threshold, with no further differentiation among p-values above the specified threshold. These major distinctions likely explain why Set Screen results were least like results from the other three methods. For additional information about similarities and differences among these methods, please see a text section ‘Pathway analysis method comparison’ in the **Methods S1**.

### Limitations

Our findings represent one attempt to seek associations between illness and groups of genes (pathways) chosen on the basis of independent evidence for likely mechanistic involvement in schizophrenia and/or bipolar disorder. The findings, as always, need to be subjected to a replication attempt, in an independent dataset of adequate size, and using the same methods and parameters. No dataset meeting these criteria is currently publically available. The ‘PGC2′ – Psychiatric GWAS/Genomic Consortium 2– schizophrenia dataset will be available in the future and is ideal for this application. Also regarding datasets, some common control subjects are included in the publically available PGC schizophrenia and bipolar disorder datasets. This should not pose a problem for interpretation of our results because shared controls would tend to make results for the two disorders more similar than they actually are. Of note, for the method-wide significant results, we found no evidence of overlapping associations between the two disorders. We cannot rule out the possibility that the non-significant trends observed across classes of glia are an example of such similarities introduced by the existence of some shared controls. However, these trends may also accurately reflect shared determinants of various psychotic disorders.

While our manuscript was under review, an independent group reported their results showing association between glial pathways and schizophrenia [38]. These results were by a different analysis that used genotype-level data from participants. This represents further evidence in support of the association observed. Combined with the other biological data on the involvement of oligocuytes in schizophrenia, the evidence is highly suggestive of a key role for these cells in determining expression of illness.

Regarding the gene sets themselves, it must be noted that pathway curation relies on incomplete information from diverse sources, of variable quality. Therefore we cannot know how well gene membership within pathways accurately reflects biologic function. However, every gene listed in a pathway is in the list because there is data supporting its inclusion. Below we discuss ways in which pathways may be refined in the future. Regarding phenotype, we could not interrogate questions regarding phenotypic heterogeneity because the GWAS phenotype was binary (i.e. schizophrenia or bipolar case vs. control). Further, were not able to select the covariates used to generate the publically available GWAS data, given that it is a static resource.

Lastly, we are aware that few genes and no gene sets are uniquely important to any cell type or cell function. Biology, by its nature, uses the same elements in diverse instances and interactive ways. The finding that particular gene sets, such as the oligodendrocyte pathway studied here, are associated with illness is strong evidence for a pathophysiologic role of these cells, especially as it is consistent with other evidence from independent studies performed using entirely different technologies. It does not rule out the likelihood that some of these same genes may be involved in other defects observed in schizophrenia, both in the CNS and peripheral cells.

### Final synopsis

Negative results from these analyses are, unfortunately, not entirely informative because it could be the case that pathways were not well specified or that power was low. However, positive findings for the Glia-Oligodendrocyte pathway in schizophrenia and, with less certainty, the Glia-Astrocyte pathway in bipolar disorder offer confirmation of neurobiological findings about these disorders, and are important targets for replication attempts and the design of new studies. Replication attempts can occur alongside efforts to perform more refined pathway analyses based on these results. For example, one could trim the associated pathway lists to exclude genes that contributed little or detracted from observed associations, and then test these ‘short lists’ in independent GWAS datasets. It is also possible to examine subsets of the genes for associations to specific features of illness, measurable by other technologies, such as white matter integrity or glial health. Mitochondrial function can be assessed in patients or in samples derived from those patients, including the possibility of examining patient-derived glial cells via induced pluripotent stem cell (IPSC) technology [39]. This approach might yield even stronger associations and more mechanistically meaningful information about the molecular and cellular processes underlying psychosis. In this way, these genomic results can lead to a wealth of complementary in vivo (eg, brain imaging) and ex vivo (eg, tissue culture) analyses on some of the contributing causes of psychoses.

## Supporting Information

Figure S1 **Distribution of 140 pathway-level p-values.**
(DOCX)

Table S1 **Gene-level p-values for the Glia-Oligodendrocyte pathway in schizophrenia dataset.**
(DOCX)

Table S2 **Schizophrenia pathway q-values by method and setting.**
(DOCX)

Table S3 **Bipolar disorder pathway q-values by method and setting.**
(DOCX)

Table S4 **Schizophrenia and bipolar disorder pathway p-values by method and setting.**
(DOCX)

Methods S1 **Supplemental Methods.**
(DOCX)

Data S1 **Entrez gene numbers for genes within each pathway.**
(DOCX)

## References

[1] Hyman SE (2012) Revolution stalled. Sci Transl Med 4: 155cm11 10.1126/scitranslmed.3003142 23052291

[2] Sullivan PF, Daly MJ, O'Donovan M (2012) Genetic architectures of psychiatric disorders: the emerging picture and its implications. Nat Rev Genet 13: 537–551 10.1038/nrg3240 22777127 PMC4110909

[3] Purcell SM, Wray NR, Stone JL, Visscher PM, O'Donovan MC, et al (2009) Common polygenic variation contributes to risk of schizophrenia and bipolar disorder. Nature 460: 748–752 10.1038/nature08185 19571811 PMC3912837

[4] Psychiatric GWAS Consortium Bipolar Disorder Working S (2011) Large-scale genome-wide association analysis of bipolar disorder identifies a new susceptibility locus near ODZ4. Nature Genetics 43: 977–983 10.1038/ng.943 21926972 PMC3637176

[5] Cross-Disorder Group of the Psychiatric Genomics Consortium, Smoller JW, Craddock N, Kendler K, Lee PH, et al (2013) Identification of risk loci with shared effects on five major psychiatric disorders: a genome-wide analysis. Lancet 381: 1371–1379 10.1016/S0140-6736(12)62129-1 23453885 PMC3714010

[6] Lips ES, Cornelisse LN, Toonen RF, Min JL, Hultman CM, et al (2011) Functional gene group analysis identifies synaptic gene groups as risk factor for schizophrenia. Molecular Psychiatry 17: 996–1006 10.1038/mp.2011.117 21931320 PMC3449234

[7] O'Dushlaine C, Kenny E, Heron E, Donohoe G, Gill M, et al (2011) Molecular pathways involved in neuronal cell adhesion and membrane scaffolding contribute to schizophrenia and bipolar disorder susceptibility. Mol Psychiatry 16: 286–292 10.1038/mp.2010.7 20157312

[8] Rajkowska G, Halaris A, Selemon LD (2001) Reductions in neuronal and glial density characterize the dorsolateral prefrontal cortex in bipolar disorder. Biol Psychiatry 49: 741–752. 11331082 10.1016/s0006-3223(01)01080-0

[9] Uranova NA, Vostrikov VM, Orlovskaya DD, Rachmanova VI (2004) Oligodendroglial density in the prefrontal cortex in schizophrenia and mood disorders: a study from the Stanley Neuropathology Consortium. Schizophr Res 67: 269–275 10.1016/S0920-9964(03)00181-6 14984887

[10] Takahashi N, Sakurai T, Davis KL, Buxbaum JD (2011) Linking oligodendrocyte and myelin dysfunction to neurocircuitry abnormalities in schizophrenia. Prog Neurobiol 93: 13–24 10.1016/j.pneurobio.2010.09.004 20950668 PMC3622281

[11] Kety SS (1950) Blood flow and metabolism of the human brain in health and disease. Trans Stud Coll Physicians Phila 18: 103–108. 14788185

[12] McBride HM, Neuspiel M, Wasiak S (2006) Mitochondria: more than just a powerhouse. Curr Biol 16: R551–560 10.1016/j.cub.2006.06.054 16860735

[13] Ben-Shachar D, Laifenfeld D (2004) Mitochondria, synaptic plasticity, and schizophrenia. Int Rev Neurobiol 59: 273–296 10.1016/S0074-7742(04)59011-6 15006492

[14] Prabakaran S, Swatton JE, Ryan MM, Huffaker SJ, Huang JT-J, et al.. (2004) Mitochondrial dysfunction in schizophrenia: evidence for compromised brain metabolism and oxidative stress. Mol Psychiatry 9: 684–697, 643. doi:10.1038/sj.mp.4001511. 10.1038/sj.mp.4001511 15098003

[15] Rezin GT, Amboni G, Zugno AI, Quevedo J, Streck EL (2009) Mitochondrial dysfunction and psychiatric disorders. Neurochem Res 34: 1021–1029 10.1007/s11064-008-9865-8 18979198

[16] Manji H, Kato T, Di Prospero NA, Ness S, Beal MF, et al (2012) Impaired mitochondrial function in psychiatric disorders. Nat Rev Neurosci 13: 293–307 10.1038/nrn3229 22510887

[17] Kato T, Kato N (2000) Mitochondrial dysfunction in bipolar disorder. Bipolar Disorders 2: 180–190 10.1034/j.1399-5618.2000.020305.x 11256685

[18] Konradi C EM (2004) Molecular evidence for mitochondrial dysfunction in bipolar disorder. Arch Gen Psychiatry 61: 300–308 10.1001/archpsyc.61.3.300 14993118

[19] Stork C, Renshaw PF (2005) Mitochondrial dysfunction in bipolar disorder: evidence from magnetic resonance spectroscopy research. Mol Psychiatry 10: 900–919 10.1038/sj.mp.4001711 16027739

[20] Cataldo AM, McPhie DL, Lange NT, Punzell S, Elmiligy S, et al (2010) Abnormalities in mitochondrial structure in cells from patients with bipolar disorder. Am J Pathol 177: 575–585 10.2353/ajpath.2010.081068 20566748 PMC2913344

[21] Javitt DC (2004) Glutamate as a therapeutic target in psychiatric disorders. Mol Psychiatry 9: 984–997 10.1038/sj.mp.4001551 15278097

[22] Coyle JT (2006) Glutamate and schizophrenia: beyond the dopamine hypothesis. Cell Mol Neurobiol 26: 365–384 10.1007/s10571-006-9062-8 16773445 PMC11881825

[23] Ongur D, Jensen JE, Prescot AP, Stork C, Lundy M, et al (2008) Abnormal Glutamatergic Neurotransmission and Neuronalglial Interactions in Acute Mania. Biol Psychiatry 64: 718–726 10.1016/j.biopsych.2008.05.014 18602089 PMC2577764

[24] Öngür D, Haddad S, Prescot AP, Jensen JE, Siburian R, et al (2011) Relationship between genetic variation in the glutaminase gene GLS1 and brain glutamine/glutamate ratio measured in vivo. Biol Psychiatry 70: 169–174 10.1016/j.biopsych.2011.01.033 21457947 PMC3125415

[25] Segrè AV, Groop L, Mootha VK, Daly MJ, Altshuler D, et al (2010) Common Inherited Variation in Mitochondrial Genes Is Not Enriched for Associations with Type 2 Diabetes or Related Glycemic Traits. PLoS Genet 6: e1001058 10.1371/journal.pgen.1001058 20714348 PMC2920848

[26] Holmans P, Green EK, Pahwa JS, Ferreira MAR, Purcell SM, et al (2009) Gene Ontology Analysis of GWA Study Data Sets Provides Insights into the Biology of Bipolar Disorder. The American Journal of Human Genetics 85: 13–24 10.1016/j.ajhg.2009.05.011 19539887 PMC2706963

[27] Lee PH, O'Dushlaine C, Thomas B, Purcell SM (2012) INRICH: interval-based enrichment analysis for genome-wide association studies. Bioinformatics 28: 1797–1799 10.1093/bioinformatics/bts191 22513993 PMC3381960

[28] Moskvina V, O'Dushlaine C, Purcell S, Craddock N, Holmans P, et al (2011) Evaluation of an approximation method for assessment of overall significance of multiple-dependent tests in a genomewide association study. Genet Epidemiol 35: 861–866 10.1002/gepi.20636 22006681 PMC3268180

[29] Psychiatric GWAS Consortium R (2011) Genome-wide association study identifies five new schizophrenia loci. Nat Genet 43: 969–976 10.1038/ng.940 21926974 PMC3303194

[30] Pickrell JK, Marioni JC, Pai AA, Degner JF, Engelhardt BE, et al (2010) Understanding mechanisms underlying human gene expression variation with RNA sequencing. Nature 464: 768–772 10.1038/nature08872 20220758 PMC3089435

[31] Gene Ontology Consortium (2004) The Gene Ontology (GO) database and informatics resource. Nucleic Acids Research 32: 258D–261 10.1093/nar/gkh036 PMC308770 14681407

[32] Matthews L, Gopinath G, Gillespie M, Caudy M, Croft D, et al (2009) Reactome knowledgebase of human biological pathways and processes. Nucleic Acids Res 37: D619–622 10.1093/nar/gkn863 18981052 PMC2686536

[33] Storey JD (2002) A direct approach to false discovery rates. Journal of the Royal Statistical Society: Series B (Statistical Methodology) 64: 479–498 10.1111/1467-9868.00346

[34] Development Core Team, R: A language and environment for statistical computing. R Foundation for Statistical Computing, Vienna, Austria. ISBN 3-900051-07-0 (2005). Available: http://www.R-project.org.Accessed: 4 Oct 2013.

[35] Storey JD, Tibshirani R (2003) Statistical significance for genomewide studies. Proc Natl Acad Sci U S A 100: 9440–9445 10.1073/pnas.1530509100 12883005 PMC170937

[36] White T, Nelson M, Lim KO (2008) Diffusion tensor imaging in psychiatric disorders. Top Magn Reson Imaging 19: 97–109 10.1097/RMR.0b013e3181809f1e 19363432

[37] Rajkowska G, Miguel-Hidalgo JJ (2007) Gliogenesis and glial pathology in depression. CNS Neurol Disord Drug Targets 6: 219–233. 17511618 10.2174/187152707780619326 PMC2918806

[38] Goudriaan A, Leeuw C de, Ripke S, Hultman CM, Sklar P, et al.. (2013) Specific Glial Functions Contribute to Schizophrenia Susceptibility. Schizophr Bull: sbt109. doi:10.1093/schbul/sbt109. 10.1093/schbul/sbt109 PMC4059439 23956119

[39] Takahashi K, Yamanaka S (2006) Induction of pluripotent stem cells from mouse embryonic and adult fibroblast cultures by defined factors. Cell 126: 663–676 10.1016/j.cell.2006.07.024 16904174

