# Supplementary material for: Pathway Analyses Implicate Glial Cells in Schizophrenia
Source: PLoS One. 2014 Feb 24;9(2):e89441. doi: 10.1371/journal.pone.0089441 (PMC3933626; doi:10.1371/journal.pone.0089441)

**Figure S1. Distribution of 140 pathway-level p-values.**

Supplemental Figure 1 demonstrates the non-uniform distribution of null p-values (negative skew in data) among the 140 tests conducted in this report. Given this distribution, we used the bootstrap method in FDR control calculations, per recommendations by Storey et al [33,35].


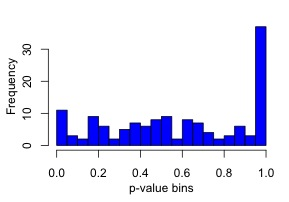

Supplement: Figure S1 — Distribution of 140 pathway-level p-values. (DOCX) [file pone.0089441.s001.docx]
