# Supplementary material for: Pathway Analyses Implicate Glial Cells in Schizophrenia
Source: PLoS One. 2014 Feb 24;9(2):e89441. doi: 10.1371/journal.pone.0089441 (PMC3933626; doi:10.1371/journal.pone.0089441)
Supplement: Table S1 — Gene-level p-values for the Glia-Oligodendrocyte pathway in schizophrenia dataset. (DOCX) [file pone.0089441.s002.docx]

**Table S1**. **Gene-level p-values for the Glia-Oligodendrocyte pathway in schizophrenia dataset.**

Analysis with MAGENTA method and top 25% setting.

| Gene name | Entrez  ID | Gene  p-value | Chr. | Start | End | SNPs  /gene | Indep.  HapMap  SNPs/  Gene | Recomb.  Hotspots  /Gene | Best  SNP  position | Best  SNP  p-value |
| --- | --- | --- | --- | --- | --- | --- | --- | --- | --- | --- |
| EIF2B4 | 8890 | 3.14E-03 | 2 | 27440722 | 27446828 | 11 | 2 | 0 | 27439659 | 2.39E-04 |
| LYN | 4067 | 1.43E-02 | 8 | 56954939 | 57086494 | 87 | 17 | 3 | 56981889 | 1.63E-04 |
| SOX6 | 55553 | 3.75E-02 | 11 | 15948370 | 16454494 | 165 | 35 | 5 | 16152464 | 8.18E-05 |
| FOXA2 | 3170 | 3.92E-02 | 20 | 22509641 | 22514101 | 19 | 1 | 2 | 22508689 | 1.52E-03 |
| SOX11 | 6664 | 4.55E-02 | 2 | 5750249 | 5758968 | 27 | 6 | 3 | 5765068 | 1.33E-03 |
| GLI3 | 2737 | 4.83E-02 | 7 | 41967072 | 42243321 | 181 | 51 | 9 | 41985224 | 2.24E-04 |
| CXCR4 | 7852 | 6.36E-02 | 2 | 136588388 | 136592195 | 18 | 4 | 0 | 136580286 | 3.09E-03 |
| LINGO1 | 84894 | 7.85E-02 | 15 | 75692423 | 75711764 | 30 | 9 | 2 | 75697257 | 2.87E-03 |
| BCL2 | 596 | 9.47E-02 | 18 | 58941558 | 59137593 | 135 | 46 | 7 | 59121016 | 1.01E-03 |
| BOK | 666 | 9.60E-02 | 2 | 242146864 | 242162226 | 13 | 5 | 2 | 242170078 | 7.76E-03 |
| CDK5 | 1020 | 1.02E-01 | 7 | 150381831 | 150385929 | 19 | 5 | 0 | 150416686 | 5.29E-03 |
| ASCL1 | 429 | 1.03E-01 | 12 | 101875581 | 101878424 | 16 | 3 | 0 | 101850400 | 6.02E-03 |
| C11orf9 | 745 | 1.10E-01 | 11 | 61276696 | 61312565 | 34 | 8 | 3 | 61247062 | 4.37E-03 |
| PPARG | 5468 | 1.51E-01 | 3 | 12304348 | 12450855 | 129 | 18 | 5 | 12456203 | 1.52E-03 |
| ID2 | 3398 | 1.51E-01 | 2 | 8739563 | 8742034 | 23 | 4 | 0 | 8746096 | 6.35E-03 |
| CDKN2C | 1031 | 1.52E-01 | 1 | 51206954 | 51212897 | 9 | 3 | 0 | 51222163 | 1.54E-02 |
| EIF2B5 | 8893 | 1.64E-01 | 3 | 185335503 | 185345793 | 24 | 3 | 0 | 185323360 | 7.68E-03 |
| HDAC10 | 83933 | 1.66E-01 | 22 | 49025739 | 49031941 | 10 | 3 | 0 | 49046788 | 1.63E-02 |
| CNTN2 | 6900 | 1.85E-01 | 1 | 203278962 | 203313761 | 61 | 11 | 4 | 203321029 | 3.10E-03 |
| DLX1 | 1745 | 1.96E-01 | 2 | 172658453 | 172662647 | 10 | 8 | 2 | 172630078 | 2.30E-02 |
| SHH | 6469 | 2.06E-01 | 7 | 155288318 | 155297728 | 5 | 8 | 2 | 155330688 | 3.16E-02 |
| SOX10 | 6663 | 2.09E-01 | 22 | 36698264 | 36710485 | 16 | 0 | 0 | 36726166 | 1.57E-02 |
| EIF2B1 | 1967 | 2.54E-01 | 12 | 122671522 | 122684200 | 27 | 6 | 2 | 122696081 | 1.40E-02 |
| SOX9 | 6662 | 2.63E-01 | 17 | 67628755 | 67634155 | 21 | 7 | 0 | 67606233 | 1.85E-02 |
| ASPA | 443 | 3.68E-01 | 17 | 3324153 | 3349450 | 37 | 21 | 2 | 3353214 | 2.66E-02 |
| DRD3 | 1814 | 4.17E-01 | 3 | 115330246 | 115380589 | 40 | 7 | 0 | 115401699 | 3.01E-02 |
| NKX2-2 | 4821 | 4.26E-01 | 20 | 21439651 | 21442664 | 12 | 1 | 0 | 21475265 | 5.51E-02 |
| ERCC2 | 2068 | 4.77E-01 | 19 | 50546685 | 50565669 | 6 | 9 | 0 | 50548889 | 1.02E-01 |
| DLX2 | 1746 | 4.92E-01 | 2 | 172672411 | 172675724 | 8 | 5 | 2 | 172700262 | 9.44E-02 |
| HES5 | 388585 | 4.99E-01 | 1 | 2450043 | 2451544 | 15 | 3 | 0 | 2455189 | 6.55E-02 |
| EXOC4 | 60412 | 5.15E-01 | 7 | 132588362 | 133401053 | 245 | 51 | 6 | 132555609 | 1.51E-03 |
| SOX8 | 30812 | 5.19E-01 | 16 | 971808 | 976980 | 17 | 7 | 2 | 944606 | 7.30E-02 |
| KCNJ10 | 3766 | 5.42E-01 | 1 | 158274656 | 158306585 | 34 | 9 | 2 | 158326896 | 5.52E-02 |
| GSTP1 | 2950 | 5.70E-01 | 11 | 67107641 | 67110700 | 17 | 4 | 2 | 67086318 | 8.17E-02 |
| HDAC11 | 79885 | 5.87E-01 | 3 | 13496823 | 13521834 | 30 | 12 | 2 | 13483455 | 7.44E-02 |
| HES1 | 3280 | 6.26E-01 | 3 | 195336627 | 195339090 | 8 | 7 | 2 | 195346502 | 1.58E-01 |
| ID4 | 3400 | 6.36E-01 | 6 | 19945595 | 19948894 | 29 | 10 | 3 | 19939797 | 6.89E-02 |
| CTNNB1 | 1499 | 6.38E-01 | 3 | 41215945 | 41256943 | 27 | 8 | 2 | 41184967 | 1.02E-01 |
| RTN4 | 57142 | 6.40E-01 | 2 | 55052830 | 55131238 | 66 | 12 | 0 | 55126791 | 5.41E-02 |
| ZNF488 | 118738 | 6.81E-01 | 10 | 47975094 | 47993874 | 9 | 5 | 2 | 47985301 | 1.80E-01 |
| NKX2-1 | 7080 | 6.82E-01 | 14 | 36055352 | 36059167 | 21 | 8 | 0 | 36065957 | 1.15E-01 |
| OLIG2 | 10215 | 6.89E-01 | 21 | 33320108 | 33323370 | 27 | 10 | 2 | 33302383 | 9.41E-02 |
| EIF2B3 | 8891 | 7.44E-01 | 1 | 45089036 | 45224869 | 53 | 7 | 0 | 45130072 | 1.12E-01 |
| CD9 | 928 | 7.45E-01 | 12 | 6179815 | 6217688 | 27 | 13 | 2 | 6146794 | 1.63E-01 |
| NOTCH1 | 4851 | 7.78E-01 | 9 | 138508716 | 138560059 | 8 | 12 | 3 | 138576033 | 2.69E-01 |
| GSN | 2934 | 7.84E-01 | 9 | 123070200 | 123134941 | 54 | 11 | 2 | 123128552 | 1.20E-01 |
| NF1 | 4763 | 7.92E-01 | 17 | 26446120 | 26728821 | 95 | 10 | 2 | 26670158 | 8.30E-02 |
| TP73 | 7161 | 8.14E-01 | 1 | 3558988 | 3640327 | 13 | 17 | 4 | 3545288 | 2.79E-01 |
| NKX6-2 | 84504 | 8.61E-01 | 10 | 134448309 | 134449527 | 18 | 8 | 2 | 134439621 | 2.75E-01 |
| EIF2B2 | 8892 | 8.80E-01 | 14 | 74539364 | 74546045 | 12 | 1 | 0 | 74553565 | 3.43E-01 |
| NKX6-1 | 4825 | 9.33E-01 | 4 | 85633459 | 85638411 | 11 | 5 | 2 | 85633765 | 5.06E-01 |
| PLP1 | 5354 | NaN | 23 | 102918094 | 102934203 | 0 | 0 | 0 | NaN | NaN |

Chr.=Chromosome, SNPs = Single Nucleotide Polymorphisms, Indep. = Independent, Recomb. = Recombination
