# Supplementary material for: Pathway Analyses Implicate Glial Cells in Schizophrenia
Source: PLoS One. 2014 Feb 24;9(2):e89441. doi: 10.1371/journal.pone.0089441 (PMC3933626; doi:10.1371/journal.pone.0089441)
Supplement: Table S2 — Schizophrenia pathway q-values by method and setting. (DOCX) [file pone.0089441.s003.docx]

**Table S2. Schizophrenia pathway q-values by method and setting.**

|  |  | **Primary Analysis** | | **Secondary Analyses** | | | | |
| --- | --- | --- | --- | --- | --- | --- | --- | --- |
|  |  | MAGENTA | | ALIGATOR | | INRICH | | SS |
|  | Genes | 95% | 75% | 95% | 75% | 95% | 75% | -- |
| **GLIA** | 146 | 0.970 | 0.627 | 0.970 | 0.970 | 0.970 | 0.970 | 0.272 |
| Glia - Oligodendrocyte | 52 | 0.970 | 0.068 | 0.970 | 0.373 | 0.970 | 0.238 | 0.970 |
| Glia - Astrocyte | 42 | 0.970 | 0.970 | 0.970 | 0.970 | 0.970 | 0.970 | 0.238 |
| **MITOCHONDRIA** | 74 | 0.970 | 0.627 | 0.970 | 0.970 | 0.970 | 0.970 | 0.970 |
| Mitochondria - Crista | 6 | 0.970 | 0.970 | 0.970 | 0.970 | 0.970 | 0.970 | 0.272 |
| Mitochondria - Distribution | 7 | 0.970 | 0.970 | 0.970 | 0.970 | 0.970 | 0.970 | 0.970 |
| Mitochondria - Fission | 12 | 0.970 | 0.970 | 0.970 | 0.970 | 0.970 | 0.970 | 0.432 |
| Mitochondria - Fission 959 | 24 | 0.970 | 0.970 | 0.970 | 0.970 | 0.970 | 0.970 | 0.421 |
| Mitochondria - Fusion | 9 | 0.970 | 0.970 | 0.970 | 0.970 | 0.970 | 0.970 | 0.776 |
| **GLUTAMATE** | 158 | 0.970 | 0.970 | 0.970 | 0.970 | 0.970 | 0.970 | 0.970 |

SS = Set Screen
