# Supplementary material for: Pathway Analyses Implicate Glial Cells in Schizophrenia
Source: PLoS One. 2014 Feb 24;9(2):e89441. doi: 10.1371/journal.pone.0089441 (PMC3933626; doi:10.1371/journal.pone.0089441)
Supplement: Table S4 — Schizophrenia and bipolar disorder pathway p-values by method and setting. (DOCX) [file pone.0089441.s005.docx]

**Table S4. Schizophrenia and bipolar disorder pathway p-values by method and setting.**

|  | **Schizophrenia** | | **Bipolar disorder** | |
| --- | --- | --- | --- | --- |
| Pathway | p-value 95% threshold | p-value 75% threshold | p-value 95% threshold | p-value 75% threshold |
| Macrophages | 0.39 | 0.41 | 0.60 | 0.79 |
| Hepatocytes | 1.00 | 0.97 | 0.72 | 0.28 |
| Lymphocytes | 0.74 | 0.89 | 0.26 | 0.78 |
