# Supplementary material for: Pathway Analyses Implicate Glial Cells in Schizophrenia
Source: PLoS One. 2014 Feb 24;9(2):e89441. doi: 10.1371/journal.pone.0089441 (PMC3933626; doi:10.1371/journal.pone.0089441)
Supplement: Methods S1 — Supplemental Methods. (DOCX) [file pone.0089441.s006.docx]

**Methods S1. Supplemental Methods**

Multiple Testing Correction

Consequently we describe raw p-values with respect to Bonferroni corrections, both method-wide (e.g. for all MAGENTA tests) and experiment-wide (i.e. for the 140 tests conducted across all methods, pathways, settings, and datasets). We also note here the specific reasons why Bonferroni correction is overly conservative for this investigation.

Method-wide Bonferroni correction for our primary analysis with MAGENTA and also for ALIGATOR and INRICH is for 40 tests (α=0.00125, given 10 pathways * 2 settings * 2 disorders). Only 20 tests were conducted with Set Screen because only one 'setting' is possible (therefore α=0.0025 for 10 pathways * 2 disorders). Bonferroni correction is overly-conservative, however, if tests are not independent. In this investigation, there are three substantial sources of non-independence: 1) correlated pathways (given superordinate and subordinate pathways); 2) common data used across different analysis settings; and 3) common design features of analysis methods. Nevertheless, as described below, our strongest finding exceeded method-wide significance and approached experiment-wide significance.

Control of FDR assumes a uniform distribution for null p-values, and our results violate this assumption, as shown in the histogram in **Supplemental Figure S1**.  This means that the methodology used is still valid, but will be conservative.  Further, Dabney and Storey note that this is a drawback of frequentist hypothesis testing, which can be overcome by using the bootstrap option available in the R package.  Per recommendation (Dabney Storey 2003) we used the pi0.method = "bootstrap" option to address this problem. Note that our q-values are still likely to be conservative because our tests were not independent.

Testing of reviewer-requested negative control pathways

Per reviewer request, we also tested three negative control pathways (lymphocytes, hepatocytes, and macrophages) for association to schizophrenia and bipolar disorder using exactly the same parameters used to test our hypothesized pathways. The three negative control pathways were constructed using a human species filter and the GO terms 'differentiation', 'regulation', and 'proliferation' for hepatocytes and GO terms 'differentiation', 'regulation' and 'activation' for lymphocytes and macrophages**.** The Entrez gene numbers for negative control pathways are given in the Data Supplement, and results for negative control pathways are provided in **Supplementary Table S4.**

Results

There was no evidence of association for any of the three negative control pathways (lymphocytes, hepatocytes, macrophages) with either schizophrenia or bipolar disorder, at either significance threshold (minimum of 12 p-values = 0.26).

**Pathway analysis method comparison**

A thorough description of methodological differences among pathway analysis methods (which can lead to some differences in results) is beyond the scope of this manuscript, but a comparative overview of the various analyses used is provided here. For further details, readers are referred to primary articles proposing each method[1–4], and a review article[5] about pathway analysis methods suitable for use with genome-wide SNP data (GWAS data).

In the service of properly orienting the reader, we note that 'pathway analysis' is a term used to describe an increasingly wide variety of analyses. In this manuscript, we chose methods designed for use with SNP-level p-values from GWAS. These pathway analysis methods are largely distinct from an established and productive field of pathway analysis devoted to gene expression data, which does not require handling of various genomic confounding factors that are specific to genetic variation data. (Note however, that there are also methods currently being developed that integrate the two types of data.) Furthermore, the GWAS pathway analysis methods used in this report are distinct from methods suitable for use with rare variants (e.g. from sequence data), which typically rely on genetic burden tests of one form or another.

Each of the methods used in this report (MAGENTA, INRICH, ALIGATOR, and Set Screen), tests whether a pathway (set of genes) shows significant enrichment of GWAS association signal to the phenotype of interest. However, there are several differences among these four methods. First, ALIGATOR, MAGENTA and INRICH are “over-representation” methods – that is, they test whether pathways contain a larger number of significant genes than would be expected by chance. SNP-level p-values are used to determine which genes are counted as significant, but are not used in the enrichment analysis itself. In contrast, Set Screen combines the p-values from all SNPs in the pathway allowing for linkage disequilibrium (LD). The approach used by Set Screen will increase power for large genes with multiple independent association signals (where the combined p-value will be more significant than the most significant single-SNP p-value) but will lose power for large genes with only one association signal (where the significance of the combined p-value will be reduced by including non-associated SNPs)

INRICH differs from ALIGATOR and MAGENTA in that it defines genomic regions based on LD with a significantly-associated SNP. These LD-independent association regions are counted as significant if they overlap with any pathway genes in the enrichment analysis. Conversely, ALIGATOR and MAGENTA define the association between genes and phenotype based on the most significantly associated SNP within that gene.

A final distinction, which separates MAGENTA from ALIGATOR, concerns the unit used for permutation in order to generate pathway level p-values. MAGENTA permutes genes whereas ALIGATOR permutes SNPs. ALIGATOR's SNP permutation procedure addresses the challenge that larger genes, by chance alone, will achieve smaller p-values. In contrast, MAGENTA addresses this challenge by 'normalizing' all genes (via multivariate linear regression) to account for confounders to gene-level p-value (such as gene size and recombination hotspots).

As we have noted in the main text, there is currently no standard way to determine which pathway analysis method is best in any case. All can give useful information. There is also no standard means to compare results, but shared findings can provide evidence of robustness in associations. Future, detailed methodological work is needed to better characterize the consequences of methodolgical differences.

1. Segrè AV, Groop L, Mootha VK, Daly MJ, Altshuler D, et al. (2010) Common Inherited Variation in Mitochondrial Genes Is Not Enriched for Associations with Type 2 Diabetes or Related Glycemic Traits. PLoS Genet 6: e1001058. doi:10.1371/journal.pgen.1001058.

2. Lee PH, O’Dushlaine C, Thomas B, Purcell SM (2012) INRICH: interval-based enrichment analysis for genome-wide association studies. Bioinforma Oxf Engl 28: 1797–1799. doi:10.1093/bioinformatics/bts191.

3. Holmans P, Green EK, Pahwa JS, Ferreira MAR, Purcell SM, et al. (2009) Gene Ontology Analysis of GWA Study Data Sets Provides Insights into the Biology of Bipolar Disorder. Am J Hum Genet 85: 13–24. doi:10.1016/j.ajhg.2009.05.011.

4. Moskvina V, O’Dushlaine C, Purcell S, Craddock N, Holmans P, et al. (2011) Evaluation of an approximation method for assessment of overall significance of multiple-dependent tests in a genomewide association study. Genet Epidemiol 35: 861–866. doi:10.1002/gepi.20636.

5. Holmans P (2010) Statistical methods for pathway analysis of genome-wide data for association with complex genetic traits. Adv Genet 72: 141–179. doi:10.1016/B978-0-12-380862-2.00007-2.
