# Supplementary material for: Pathway Analyses Implicate Glial Cells in Schizophrenia
Source: PLoS One. 2014 Feb 24;9(2):e89441. doi: 10.1371/journal.pone.0089441 (PMC3933626; doi:10.1371/journal.pone.0089441)
Supplement: Data S1 — Entrez gene numbers for genes within each pathway. (DOCX) [file pone.0089441.s007.docx]

**Data S1**. Entrez gene numbers for genes within each pathway.

Hypothesized Pathways

Mitochondria 10059 10131 1020 10730 10845 10891 10939 11331 1152 120892 1352 1600 168620 172 1723 20112 201164 205564 22906 2309 23095 24752 267012 27185 27429 30968 3799 4131 4205 449520 4534 4729 4780 4899 4976 5018 51024 51537 5245 5292 54332 54471 54518 54708 54927 55154 55288 55333 55486 5562 5563 55669 5568 572 57534 578 581 598 6183 64422 64850 65018 66008 664 665 6687 7284 7341 79594 8192 8871 89941 983 9927

Glutamate 10165 1020 10368 10369 10550 10755 10815 11230 112755 116443 116444 1201 1268 134 135 1373 1392 1393 144193 149111 1499 154 1612 162417 1741 1742 1808 1812 1813 2055 2099 22871 22941 22999 23236 23542 23657 246213 254263 2571 2572 27091 27092 27165 2729 2730 2744 2746 2747 2752 2776 2778 2805 2806 2890 2891 2892 2893 2894 2895 2897 2898 2899 2900 2901 2902 2903 2904 2905 2906 2908 291 2911 2912 2913 2914 2915 2916 2917 2918 320 3351 3356 351 3553 3766 435 4645 4735 478 4889 491 4983 4988 5021 5023 50488 5071 50944 51166 53358 54407 54413 551 552 553 5566 5625 5649 5663 57030 57084 5743 57555 58510 5864 5867 5923 59283 59284 627 63908 6505 6506 6507 6509 6511 6512 6616 6751 6804 6812 6844 6857 6898 7124 7143 7200 729993 7349 7442 773 7915 79751 79953 80262 85358 8604 8775 8851 887 9114 9378 9454 9455 9456 947 9971

Glia 1020 1021 10215 1031 10319 10397 10683 118738 1270 1462 1499 1583 163175 1745 1746 1812 1814 183 1840 1959 1967 200350 2043 2064 2065 2068 2119 2147 2185 2247 2250 2255 22809 2297 2298 2306 23405 23529 2621 2670 27022 27339 2737 286380 28999 2934 2950 301 30812 3145 3170 3280 3398 3400 3479 349334 3766 388585 3913 3976 4040 4067 429 430 4323 443 4504 4664 4665 4692 4763 4771 4781 4821 4825 4851 4908 4916 5048 5076 51176 5156 5270 5324 5354 5453 5454 5468 55553 5583 5584 5649 566 57142 58158 596 5970 60412 6347 6348 637 64398 6469 6497 650 653404 653427 6647 6657 6659 666 6662 6663 6664 6774 6886 6900 7042 706 7080 7101 716 7161 729230 745 7804 7852 79006 79727 79885 8091 83933 84504 84894 8521 8890 8891 8892 8893 8929 9241 92737 928 9639 983 9921

GliaAstrocytes 1021 10319 10683 1270 1812 183 2043 2147 22809 23529 2670 27339 30812 3145 3280 3398 3400 388585 3913 3976 4323 4504 4763 4821 4851 4916 5270 5454 6347 6348 6469 650 6662 6774 6886 6900 7101 729230 8091 8521 8893 9241

GliaOligocytes 1020 10215 1031 118738 1499 1745 1746 1814 1967 2068 2737 2934 2950 30812 3170 3280 3398 3400 3766 388585 4067 429 443 4763 4821 4825 4851 5354 5468 55553 57142 596 60412 6469 666 6662 6663 6664 6900 7080 7161 745 7852 79885 83933 84504 84894 8890 8891 8892 8893 928

MitochondriaCrista 125965 1583 2230 4067 4976 8165

MitochondriaDistribution 120892 168620 4205 4534 55154 55333 8871

MitochondriaFission 10059 1352 1723 449520 4976 51024 51537 54708 581 64422 664 79594

MitochondriaFission_plus 10059 1352 1723 449520 4976 51024 51067 51537 5442 54708 55157 56652 581 6182 64216 64422 664 6832 7019 79594 79736 7978 80222 87178

MitochondriaFusion 201164 4976 54471 54927 55669 578 581 664 9927

Reviewer-requested negative control pathways

Macrophages 7099 1437 1907 6556 7096 23210 22918 3725 566 199 5581 22904 7305 6850 148022 1524 64127 51284 10062 3566 671 7032 80149 133396 9173 3566 90865 7057 644745 3929 3596 7474 124912 79155 6622 8399 4282 6095 6542 23729 3684 2624 8563 714 3623 3624 5771 9370 140685 841 1435 1436 652 4318 7422 6688 1050 643336 6689 1053 159296 139716 5196 5925 796 3059 3976 5578 3398 8737

Hepatocytes 3087 6928 2487 79733 1543 1373 5629 144455 301 7471 55897 55294 249 7128 5469 7429 8829 6997 6998 200958 5770 5771 4233 3082 3175 5294 3625

Lymphocytes 100 101 8546 8943 558 572 55024 604 652 684 100129681 695 6363 4064 914 939 973 941 9308 942 1137 1141 1380 1493 643 1840 8320 2322 2323 2302 50943 202309 2621 732446 2625 55340 2736 2737 9759 10014 9734 3142 3329 345041 643300 644745 645548 23308 3398 3440 3456 3458 56832 280655 3476 3549 10320 22806 3592 3593 3594 3600 3601 3606 8809 8808 3558 59067 50615 51561 149233 3559 3565 3566 3569 3574 3575 3623 3624 3635 3481 3630 723961 3659 3707 3718 3811 3813 22914 7462 54900 3932 10288 4276 4277 931 4602 3071 9437 84807 79576 441478 55795 5201 8993 114770 114771 57115 5293 4860 639 5579 5590 5788 5914 149041 8767 91543 9092 54440 6422 6469 8935 6504 57823 9655 6776 6777 30968 6850 7048 7099 7292 200081 80329 80328 79465 8876 7535 22890 84524
